# SINE (selective inhibitor of nuclear export) – translational science in a new class of anti-cancer agents

**DOI:** 10.1186/s13045-014-0067-3

**Published:** 2014-10-04

**Authors:** John Gerecitano

**Affiliations:** Department of Medicine, Memorial Sloan Kettering Cancer Center, 1275 York Avenue, New York, NY 10065 USA; Weill Cornell Medical College, New York, NY 10065 USA

## Abstract

Regulation of protein trafficking between the nucleus and cytoplasm represents a novel control point for antineoplastic intervention. Several proteins involved with cellular growth and survival depend on precise and timely positioning within the cell to fulfill their functions, and the nuclear membrane defines one of the most important compartmental barriers. Chromosome Region Maintenance 1, or exportin-1 (CRM1/XPO1), is involved with the export of more than 200 nuclear proteins, and has intriguingly been shown to have an increased expression in several tumor cell types. Selinexor (KPT-330) is a first-in-class selective inhibitor of nuclear export (SINE) to be developed for clinical use. Preclinical data has demonstrated antineoplastic activity of SINE compounds in many human solid and hematologic malignancies. The clinical development of Selinexor provides an excellent model for translational research.

## SINE – translational science in a new class of anti-cancer agents

There are many ways in which neoplastic cells can gain a selective survival advantage over their non-cancerous counterparts. While traditional chemotherapeutics target the end result of cell division caused by many transformative events, more recent ‘targeted therapies’ have aimed to selectively block specific aspects of neoplastic cells that allow them to compete for resources and grow without restraint. For the most part, targeted therapies include those that 1) block external signals that can lead to cell growth, 2) interrupt intracellular signal transduction or enzymatic pathways that mediate growth and/or metabolism within neoplastic cells, or 3) thwart the cancer cell’s ability to evade signals and intracellular process such as apoptosis that would otherwise lead to self destruction. Many of these therapeutic strategies target cancer cells through antibody recognition or small molecule interaction with specific proteins that mediate pro-survival processes. Other strategies (such as inhibition of the proteasome, histone deacetylases, DNA methylation and heat shock proteins) seek to more globally manipulate the expression and longevity of the proteins that lead to the selective advantage of tumor cells.

Regulation of protein trafficking between the nucleus and cytoplasm represents a novel control point for antineoplastic intervention. Several proteins involved with cellular growth and survival depend on precise and timely positioning within the cell to fulfill their functions, and the nuclear membrane defines one of the most important compartmental barriers. Although molecules smaller than 40 kDa can passively move between the nucleus and cytoplasm, larger molecules depend on active transport mediated by karyopherin chaperones [1]. Once such karyopherin that is exclusively engaged in nuclear export is Chromosome Region Maintenance 1, or exportin-1 (CRM1/XPO1). CRM-1 is involved with the export of more than 200 nuclear proteins, and has intriguingly been shown to have an increased expression in several tumor cell types [2–9]. The selective advantage this confers may relate to the proteins exported by CRM1, many of which (e.g. p53, p21, p27, pRb, FOXO, IkB) behave as tumor suppressors when localized to the nucleus, thus relieving inhibition of cellular growth and survival when they are exported to the cytoplasm [1].

Selinexor (KPT-330) is a first-in-class selective inhibitor of nuclear export (SINE) to be developed for clinical use. By interacting directly with CRM1, this drug prevents its interaction with potential cargo and traps in the nucleus proteins that would otherwise have been destined for export to the cytoplasm. Preclinical data has demonstrated antineoplastic activity of SINE compounds in many human solid and hematologic malignancies including renal cell carcinoma [10], mantle cell lymphoma (MCL) [8], chronic lymphocytic leukemia (CLL) [11], multiple myeloma (MM) [9], melanoma [12], acute myeloid leukemia (AML) [13] and non-Hodgkin lymphoma (NHL) [14]. Recent work has also shown that SINE compounds increase CRM1’s proteasomal degradation, increase nuclear retention of p53 and Foxo, and lead to increased apoptosis in prostate cancer cell lines [15].

In this issue of *Journal of Hematology & Oncology*, Gravina *et al*. explore mechanisms by which SINE compounds can affect the growth and spread of prostate cancer tumor lines [16]. The authors use tumor lines with different metastatic potentials and tropisms, and test tumor biology within different *in vivo* models. In one model, the authors orthotopically transplanted PCa cells into the prostate glands of SCID mice. Direct growth of the orthotopic tumor and extension into the peritoneum were both inhibited by KPT-330 given at two different dose levels and dosing schedules. In two models of bone metastasis, the authors injected PCa cells into the heart or directly into the tibiae of SCID mice. Both models showed decreased growth of osteolytic lesions upon treatment with SINE, as well as a decrease in markers of bone resorption and increased survival of the treated mice. To further demonstrate the antimetastatic potential of these inhibitors, the authors demonstrated decreased migratory potential across Boyden chambers, decreased metalloprotease production and decreased survival of cells detached from their microenvironment when treated with Selinexor. Perhaps even more intriguingly, they found that Selinexor can inhibit markers of angiogenesis and osteoclast differentiation induced by contact with media conditioned by PCa cells. In sum, these results suggest a multitude of ways that SINE can affect the biology and natural history of prostate cancer, from direct inhibition of cell growth to alterations in the microenvironment that mitigate not only metastasis, but destructive lesions fostered by nearby tumor growth.

The clinical development of Selinexor provides an excellent model for translational research. In 2012, first-in-man phase 1 trials of KPT-330 were begun in both solid (NCT01607905) and hematologic (NCT01607892) malignancies (www.clinicaltrials.gov), and early reports of these trials are showing activity in various tumor types. At the 2013 annual meeting of the American Society of Hematology (ASH), updated results from the hematologic malignancy phase 1 trial demonstrated activity in patients with multiple myeloma, Waldenstrom’s macroglobulinemia, AML, CLL and various subtypes of NHL [14,17,18]. Paired pre-and post-treatment biopsies obtained during these trials have shown increased intranuclear retention of tumor suppressor proteins in several patients: e.g. increased nuclear localization of p53 in a patient with MCL, retention of p53, FOXO3 and IkB in MM and of FOXO1, pRB, survivin and IkB in AML cells. Data from the solid malignancy trial was presented at the annual meeting of the American Society for Clinical Oncology (ASCO) in 2014, and showed decreases in disease burden in patients with colorectal, head & neck, ovarian, cervical, prostate and other malignancies including partial remissions in 3 tumor types [19]. Once again, paired biopsies in this trial were able to identify increased localization of tumor suppressor proteins (TSPs) including p53, p27, IkB, BRCA2 and FOX03 in various tumor types. Major side effects across these trials have included nausea, vomiting, diarrhea, thrombocytopenia and weight loss (the last of which caused the early clinical termination of a prior nuclear export inhibitor, Leptomycin B [20]), and the optimal dosing schedule has not yet been determined.

It will be very interesting to follow prostate patients on the phase 1 and subsequent trials that allow their inclusion. The model presented in this issue of JHO might predict numerous potential benefits for such patients. If the effects in humans reflect the *in vivo* experience presented here, we might expect to see direct decreases in osseous metastasis as well as decreases in lytic lesions caused by disruption of osteoclastogenesis in the microenvironment. This could alleviate one of the most vexing adverse effects of PCa – the pain that comes from bony lesions – even if tumor burden is not measurably decreased. Of course, the results reported also portend hope of improving the total disease burden, but it is too early to tell how deep or durable these responses can be with single-agent therapy. While these are compelling results for a new class of agents, we anxiously await results that will guide the optimal dosing schedule, mitigation of SINE-induced adverse effects, and best combination treatments to pursue.
